# Supplementary material for: Polyketide synthase gene clusters in the ericoid mycorrhizal fungus Oidiodendron maius: genome-wide prediction, expression profiling, and functional characterization of knockout mutants
Source: Front Plant Sci. 2026 Sep 16;17:1924430. doi: 10.3389/fpls.2026.1924430 (PMC13623903; doi:10.3389/fpls.2026.1924430)
Supplement: Supplementary file 1 [file SupplementaryFile1.zip › Supplementary Materials/Supplementary Folder 1.zip!/Oidma1_AssemblyScaffolds_Repeatmasked/knownclusterblast/cluster6/jgi.p_Oidma1_108948_mibig_hits.html]

| MIBiG Protein | Description | MIBiG Cluster | MiBiG Product | % ID | % Coverage | BLAST Score | E-value |
| --- | --- | --- | --- | --- | --- | --- | --- |
| BAV32147.1 | geranylgeranyl\_diphosphate\_synthase | BGC0001373 | Sordarin\_biosynthetic\_gene\_cluster | 40 | 55.4035567715 | 259 | 9.8e-69 |
| ctg1\_orf003 |  | BGC0000688 | Copalyl\_diphosphate\_biosynthetic\_gene\_cluster | 40 | 41.0396716826 | 222 | 1.3e-57 |
| CAA75568.1 | geranylgeranyl\_diphosphate\_synthase | BGC0001270\_c3 | Gibberellin\_A3\_/\_gibberellin\_A1\_/\_gibberellin\_A7\_/\_gibberellin\_A4\_/ | 51 | 29.6853625171 | 221 | 2.9e-57 |
| AAK11531.1 | PaxG | BGC0001082 | Paxilline\_biosynthetic\_gene\_cluster | 39 | 45.4172366621 | 217 | 5.6e-56 |
| AAT65717.1 | geranylgeranyl\_diphosphate\_synthase | BGC0000629 | Aflatrem\_biosynthetic\_gene\_cluster | 40 | 40.3556771546 | 216 | 1.2e-55 |
| AAW88510.1 | geranylgeranyl\_diphosphate\_synthase | BGC0001081 | Lolitrem\_biosynthetic\_gene\_cluster | 39 | 39.5348837209 | 211 | 3.1e-54 |
| BAD29970.1 | geranylgeranyldiphosphate\_synthase | BGC0000676 | Aphidicolin\_biosynthetic\_gene\_cluster | 39 | 42.1340629275 | 211 | 3.1e-54 |
| ctg1\_orf5 |  | BGC0000685 | Brassicicene\_C\_biosynthetic\_gene\_cluster | 40 | 41.7236662107 | 208 | 3.4e-53 |
| CAP07654.1 | geranylgeranyldiphosphate\_synthase | BGC0001269\_c2 | Gibberellin\_A3\_/\_gibberellin\_A1\_/\_gibberellin\_A7\_/\_gibberellin\_A4\_/ | 38 | 41.5868673051 | 205 | 2.9e-52 |
| CBF85177.1 | conserved\_hypothetical\_protein | BGC0000673 | Ent-pimara-8(14),15-diene\_biosynthetic\_gene\_cluster | 32 | 55.4035567715 | 195 | 3e-49 |
| AAK11529.1 | PaxC | BGC0001082 | Paxilline\_biosynthetic\_gene\_cluster | 30 | 29.8221614227 | 95 | 3.2e-19 |
